# Perception on medical ethics in teaching among medical students in India

**DOI:** 10.6026/97320630018613

**Published:** 2022-07-31

**Authors:** Ashwini K Shetty, Geetha S

**Affiliations:** 1 Department of physiology, Sri Devaraj URS medical college, Sri Devaraj Urs Academy of Higher Education and Research (deemed to be university), Tamaka, Kolar 563101, Karnataka, India;

**Keywords:** medical ethics, students, perception

## Abstract

The importance of teaching medical ethics has emerged as a priority in recent decades. It is of interest to document data on the perceptions of the medical students on teaching professionalism and medical ethics during the foundation course using a validated questionnaire. The Cross sectional study was conducted a Medical college in South India consisted of 150 first year MBBS Students. We received 133 responses ,40% of the students agreed that medical ethics is just a common sense, Many students (80%) agreed that the topics taught during these medical ethics sessions were relevant , easy to comprehend, teaching learning methods adopted were appropriate and they were able to participate and engage in the teaching learning activity. Majority felt that the sessions created awareness about the ethical dilemmas that might arise during patient encounters and these sessions will help them to give a justified response and agreed that these sessions made them understand the foundations of philosophical, social and legal aspects of medical ethics and also motivated them to learn more about this medical ethics .Education in medical ethics is important to practise professionally and will improve their personality skills. Suggestions given to improve ethics teaching were to increase case based discussions, reflections by the senior faculty, movie demonstrations. Students identified importance of ethics education in current day and also favoured interactive methods of teaching for the delivery of ethics related competency.

## Background:

The importance of teaching medical ethics to students was recognised a long time ago, but it is only in the last few decades that it has emerged as a priority. Ethics teaching has become integral part of the core curriculum in most medical schools in western countries. [1] In India, ethics education was never a part of formal curriculum but imparted as hidden curriculum. National Medical Commission (NMC), in its regulations on graduate medical education 2018, has elaborated the roles of the Indian medical graduate (IMG) as a clinician, leader and member of the health care team, communicator, and lifelong learner and with attributes of professionalism and ethics. [2] To fulfill the role of professional with ethical behavior the NMC has rolled out the Attitude, Ethics and Communication (AETCOM) module [3] which is a case-based module offers a framework of competency-based learning in the domains of medical ethics. Before implementing the AETCOM, the NMC introduced a mandatory training for the faculty of medical colleges through its nodal and regional centers. The NMC also proposed to teach professional development and ethics during the foundation course for duration of 40 Hours. [4] The challenges faced were shortage of faculty trained in ethics teaching to deliver the contents appropriately. Other challenges that we faced were creating resources and teaching materials to deliver these competencies and the assessment of these competencies. This study is aimed at studying the perceptions of the medical students on teaching professionalism and medical ethics during the foundation course. These perceptions will help us improve our teaching skills; provide inputs on the appropriateness of the teaching learning materials used and the usefulness of the sessions imparted. Therefore, it is of interest to document data on the perception of medical students on medical ethics teaching using a validated questionnaire.

## Methodology:

The Cross sectional study was conducted a Medical college in South India consisted of 150 first year MBBS Students. Institutional ethical clearance was obtained and Informed consent was taken by the participants of the study. The data was collected using in house developed validated questionnaire which included 13 items. 12 out of 13 items included questions on need of the ethics sessions, usefulness of the session, comprehension, appropriateness of teaching learning methods used, evoking interest and improved self-directed learning on these topics. The responses were recorded using Likert scale with rating 5 for Strongly Agree(SA), 4 for Agree(A),3 for Neutral (N), 2 for Disagree(D) and 1 for Strongly Disagree(SD) .One item in the questionnaire was open ended question which sought opinion on how to improve the effectiveness of the session delivered. The questionnaire was pilot tested on 30 students who are at the end of first year MBBS who were exposed to ethics teaching .The Cronbach's alpha was 0.8 which showed good internal consistency of the questionnaire .The questionnaire were shared with the students after delivering 40 sessions on professionalism and ethics during their foundation course. During these sessions on ethics the students were introduced to the concept of professionalism, communication skills that have to be developed by the students for future encounters with patient care. There were taught about the value of honesty, mutual respect, accountability and Integrity. They were also sensitised about the concept of empathy, altruism, pursuit of excellence .Cultural competence and disability competencies were imparted to the students. The sessions were delivered using various teaching learning methods like didactic lectures, movie screening, group discussion, reflections, role plays and panel discussions. The responses were collected using Google forms and the data thus obtained was entered in excel sheet and expressed using descriptive statistics like mean and standard deviation.

## Results:

We received 133 responses out of 150. Although 40% of the students agreed that medical ethics is just a common sense (Figure 1 - see PDF). Many students (80%) as represented in Figure 2 & Figure 3(see PDF) agreed that the topics taught during these medical ethics sessions were relevant , easy to comprehend, teaching learning methods adopted were appropriate and they were able to participate and engage in the teaching learning activity Majority (88%) of the students felt that the sessions created awareness about the ethical dilemmas that might arise during patient encounters and these sessions will help them to give a justified response as represented in Figure 4(see PDF). Majority also felt that education in medical ethics is important to practise professionally and will improve their personality skills as depicted in Figure 5(see PDF). Students were asked for any recommendations to improve the effectiveness of the sessions students recommended decreasing the didactic lectures and increasing the interactive methods like reflection by senior faculty, sharing of life experiences, case based discussion, movie demonstrations.

## Discussion:

The expedition of ethics being inculcated in medical ethics curriculum is interesting and rough [5] Teaching ethics is distinct in the medical curriculum and cannot be taught like any other subjects. However this problem has been eased by the NMC coming up with AETCOM which clearly mentions the competencies, the teaching learning method to be adopted and the assessment strategies that needs to be adopted. Ethics teaching has been new to the medical educators as it was always a part of hidden curriculum hence students expectations and perceptions on ethics teaching might be an important contributing factor to improve the efficacy and understanding of ethics sessions. The study revealed the openness of the students to learn medical ethics, their eagerness to learn more about medical ethics and also to inculcate this knowledge in future practice .This is further enhanced by the significant percentage of the students agreeing upon that ethics teaching is crucial in improving their communicative competencies, help them practice professionally and also contributed to their personal development. These findings are consistent with other Indian researchers. [6,7] The sessions on ethics created awareness about the ethical dilemmas that might arise during patient encounters and these sessions will help them to give a justified response. These findings are similar to a study which revealed that teaching of ethics will have a great impact to sensitise students about its relevance and they would think more deeply of the ethical dilemmas that will be encountered. [1, 8] Study revealed that the topics taught during the medical ethics sessions were relevant, easy to comprehend, teaching learning methods adopted were appropriate and they were able to participate and engage in the teaching learning activity .As with other studies [9] interactive methods of teaching like case based discussions, group discussions were favoured by the students. Consistent with other studies [10] students have not recognised lectures as a teaching learning method to deliver ethics related competencies. These findings act as faculty guide for medical educators as what to avoid when delivering ethics related competencies. Knowledge sharing in ethics can be done by mixture of methods that favour process of cognition decision making, critical thinking and reflection. Adequate knowledge of medical ethics can be shared by combination of different teaching learning methods like lectures, small group discussion, role play, case based discussion and also observations during clinical rounds. [11-13].

## Conclusions:

Students identified the importance of ethics education in current day and also favoured interactive methods of teaching for the delivery of ethics related competency.
